# Central Compensation in Auditory Brainstem after Damaging Noise Exposure

**DOI:** 10.1523/ENEURO.0250-18.2018

**Published:** 2018-08-17

**Authors:** Katrina M. Schrode, Michael A. Muniak, Ye-Hyun Kim, Amanda M. Lauer

**Affiliations:** 1 Department of Otolaryngology, Johns Hopkins School of Medicine, Baltimore, MD 21205; 2 Hearing Research, Garvan Institute of Medical Research, Sydney, NSW 2010, Australia; 3 St Vincent’s Clinical School, UNSW Sydney, Sydney, NSW 2052, Australia

**Keywords:** Auditory, brainstem, compensation, hyperactivity

## Abstract

Noise exposure is one of the most common causes of hearing loss and peripheral damage to the auditory system. A growing literature suggests that the auditory system can compensate for peripheral loss through increased central neural activity. The current study sought to investigate the link between noise exposure, increases in central gain, synaptic reorganization, and auditory function. All axons of the auditory nerve project to the cochlear nucleus, making it a requisite nucleus for sound detection. As the first synapse in the central auditory system, the cochlear nucleus is well positioned to respond plastically to loss of peripheral input. To investigate noise-induced compensation in the central auditory system, we measured auditory brainstem responses (ABRs) and auditory perception and collected tissue from mice exposed to broadband noise. Noise-exposed mice showed elevated ABR thresholds, reduced ABR wave 1 amplitudes, and spiral ganglion neuron loss. Despite peripheral damage, noise-exposed mice were hyperreactive to loud sounds and showed nearly normal behavioral sound detection thresholds. Ratios of late ABR peaks (2–4) relative to the first ABR peak indicated that brainstem pathways were hyperactive in noise-exposed mice, while anatomical analysis indicated there was an imbalance between expression of excitatory and inhibitory proteins in the ventral cochlear nucleus. The results of the current study suggest that a reorganization of excitation and inhibition in the ventral cochlear nucleus may drive hyperactivity in the central auditory system. This increase in central gain can compensate for peripheral loss to restore some aspects of auditory function.

## Significance Statement

Noise exposure can cause significant damage to the peripheral auditory system. Previous work has shown that the dorsal cochlear nucleus, inferior colliculus, auditory thalamus, and auditory cortex become hyperactive following damage to the peripheral auditory system, which may compensate for decreased sensory input. Here we show that after noise exposure, hyperactivity develops in the auditory brainstem as a result of reorganization of excitation and inhibition in the ventral cochlear nucleus. The compensatory plasticity observed at this early stage of the primary auditory pathway may contribute to the hyperactivity reported at later stages. Further, we show that this hyperactivity can compensate for reduced sensory input to partially restore some aspects of auditory function.

## Introduction

Deafness can have detrimental consequences on synapse morphology and function of the central auditory system (Gravel and Ruben, 1996; Shepherd et al., 2006; Muniak et al., 2013a). Substantial gaps remain in our knowledge of the central effects of acquired hearing loss. The goal of the present study was to link central changes in neural reorganization with changes in brainstem physiology and perception in an animal model of bilateral noise-induced hearing loss.

One of the most common causes of acquired hearing loss is damaging noise exposure. Many studies have detailed the consequences of noise exposure on the anatomy, physiology, and function of the inner ear (Schmiedt, 1984; Saunders et al., 1985; Henderson et al., 2006). Noise exposure can damage hair cells, supporting cells, auditory afferent dendrites, spiral ganglion neurons (SGNs), and the stria vascularis, all resulting in reduced afferent input to the central auditory system. A growing literature suggests that increased neural activity develops in central auditory structures including the dorsal cochlear nucleus (DCN), inferior colliculus, thalamus, and auditory cortex following sound exposure (Salvi et al., 2000; Seki and Eggermont, 2003; Ma et al., 2006; Brozoski et al., 2007; Kaltenbach, 2007; Bauer et al., 2008; Izquierdo et al., 2008; Shore et al., 2008; Longenecker and Galazyuk, 2011; Middleton et al., 2011; Vogler et al., 2011; Barker et al., 2012; Dehmel et al., 2012b; Gold and Bajo, 2014; Kalappa et al., 2014). The predominant hypothesis is that central hyperactivity acts as a gain control to compensate for the reduced afferent input from the ear (Potashner et al., 1997; Kaltenbach et al., 2000; Milbrandt et al., 2000; Salvi et al., 2000; Brozoski et al., 2002; Schaette and McAlpine, 2011).

There is some evidence that both spontaneous (Vogler et al., 2011; Robertson et al., 2013) and evoked (Boettcher and Salvi, 1993; Cai et al., 2009) neural activity are enhanced in the ventral cochlear nucleus (VCN) following noise exposure. Other studies have found evidence for hyperactivity in the brainstem using auditory brainstem responses (ABRs; e.g. Hickox and Liberman, 2014; Lowe and Walton, 2015; Möhrle et al., 2016). The first peak of the ABR, generated by the auditory nerve, is reduced after noise exposure. Later ABR waves (2–5 in animals, III to V in humans) are primarily generated by bushy cells of the VCN and bushy cell–driven pathways (Melcher and Kiang, 1996). Following sound exposure, these central waves, or their amplitudes relative to wave 1, are increased, indicating hyperactivity (Hickox and Liberman, 2014; Lowe and Walton, 2015; Möhrle et al., 2016).

Hyperactivity observed in the later ABR waves presumably reflects an imbalance of excitation and inhibition, contributing to relatively larger neural responses in the VCN and upstream auditory brainstem nuclei compared to the auditory nerve. This imbalance could be due to increased activity in excitatory synapses and/or reduced inhibitory activity. Previous work has found evidence for both. Glutamatergic release and receptor gene expression in the VCN show long-term increases after unilateral exposure (Muly et al., 2004; Dong et al., 2010). On the other hand, inhibitory GABAergic and glycinergic receptor genes are downregulated shortly after unilateral sound exposure but upregulated over a longer time scale (Dong et al., 2010). These changes have not been investigated for bilateral noise exposures.

In the present study, we investigated the perceptual, physiologic, and anatomic consequences of bilateral noise exposure in mice. We used a combination of ABRs and immunohistochemistry to determine if the VCN exhibits sound-evoked hyperactivity following sound exposure and measured tone detection and acoustic startle reflex (ASR) to test whether this hyperactivity could functionally compensate for reduced afferent input to support detection of and reactivity to sounds. Compared to sham-exposed mice, noise-exposed mice exhibited increased hyperactivity in the brainstem and an imbalance between excitatory and inhibitory presynaptic protein expression, but showed only small elevations in perceptual thresholds and enhanced reactivity to sound. Our results suggest that central hyperactivity can compensate for decreased sensory input to restore some auditory function.

## Materials and Methods

### Subjects

Subjects were young adult male and female CBA/CaJ mice bred in our colony from founders originally obtained from Jackson Laboratory (stock #000654). The numbers used in each procedure are detailed in Table 1. Animals were bred and housed in a quiet, low-traffic room to minimize extraneous noise exposure (Lauer et al., 2009). All procedures were approved by the Johns Hopkins University Animal Care and Use Committee and performed in accordance with the Guide for the Care and Use of Laboratory Animals.

**Table 1.** Numbers of CBA/CaJ mice of used for each procedure

Procedure	Total mice	Sham mice (F; M)	Noise mice (F; M)	Age
ABR	82	41( 21; 20)	41 (22; 19)	10 weeks
GAD65 labeling	12	7 (3; 4)	5 (2; 3)	2.5–5 months
VGLUT1 labeling	12	6 (3; 3)	6 (3; 3)	2.5–5 months
SGN counts	18	11 (5; 6)	7 (4; 3)	2.5–5 months
CTBP2 labeling	8	3 (1; 2)	5 (1; 4)	2.5–5 months
ASR	21	10 (6; 4)	11 (4; 7)	2.5–5 months
CLS	14	14 (7; 7)	5 (2; 3)	2.5–5 months

### Noise exposures

Noise exposures and sham exposures were performed on 6-week-old mice. Home cages were placed in the room containing the apparatus 30 min before commencement of exposure to allow subjects time to acclimate to the new surroundings. For both noise and sham exposure conditions, awake mice were selected at random and placed in a small wire cage mounted inside a small sound-attenuating booth [Industrial Acoustic Company (IAC)] below two speakers (TW57; Pyramid Audio). Broadband noise was generated using Matlab (MathWorks) and broadcast via a processor [RX6; Tucker-Davis Technologies (TDT)] and an amplifier (CH1; Crown Harman). We calibrated the noise to 100 dB SPL at the location of the cage before exposure using a half-inch free-field microphone and Z-weighting (SoundTrack LxT, Larson Davis). Mice were exposed to broadband noise (2–50 kHz) for 2 hours while the cage was gradually rotated within the sound field. Animals undergoing sham exposure conditions (controls) were placed in the rotating cage inside the sound booth for 2 hours, but no noise was presented. The same experimenter, a young adult female, performed all exposures. Exposures occurred during the hours of 8:00 am and 6:00 pm with the booth lights turned on so as not to disrupt the mice’s circadian cycle.

### Auditory brainstem response

We recorded ABRs 1 month after exposure to measure auditory sensitivity and determine if the auditory brainstem exhibited hyperactivity. Procedures were similar to those described by McGuire et al. (2015) and Lauer (2017). Mice were anesthetized with 100 mg/kg ketamine and 20 mg/kg xylazine and placed on a heating pad inside a small sound-attenuating chamber (IAC) lined with Sonex acoustic foam to reduce acoustic reflections. Mice were placed in front of a speaker (FT28D; Fostex) with the speaker positioned 30 cm from the vertex of the skull. We monitored body temperature via a rectal probe and maintained it at 36 ± 1°C. Subcutaneous platinum needle electrodes were placed over the left bulla and at the vertex of the skull, and a ground electrode was inserted into the leg muscle. The electrodes were attached to a preamplifier leading to an amplifier (ISO-80; World Precision Instruments).

Stimulus generation, presentation, and response acquisition were controlled using custom Matlab-based software, a TDT RX6, and a PC. Stimuli consisted of clicks (0.1-ms square wave pulse of alternating polarity) and 5-ms tones at frequencies of 6, 8, 12, 16, 24, and 32 kHz (0.5-ms onset/offset), generated with a sampling frequency of 195 kHz, and presented at a rate of 20/s. We calibrated stimuli using a quarter-inch free-field microphone (type 4939; Brüel and Kjær) placed at the location of the mouse’s head and custom Matlab-based software. Responses were sampled at 9.5 kHz, bandpass filtered from 300 to 3000 kHz, and averaged over 300 stimulus repetitions. We tested clicks first, to verify electrode placement and the presence of a clearly observable response, and then tested tone stimuli in random order. We presented a given stimulus at descending levels starting at 85–105 dB (depending on frequency) until a threshold was reached. Threshold was defined as the sound level at which the ABR peak-to-peak (any peak) amplitude was two standard deviations above the average baseline amplitude during the period of the recording when no sound stimulus was present. The amplitudes of the ABR and baseline were calculated within 8-ms windows beginning 2 ms after stimulus onset and ∼20 ms after onset, respectively. We manually measured amplitudes of peaks 1 through 4 offline using custom software. Peak 5 was not reliably detected in our recordings, consistent with reports from others (Zheng et al., 1999), so we did not measure or analyze it further. Testing lasted ∼40–60 min; mice were returned to their home cages following testing and monitored until recovery.

### Acoustic startle reflex

We used ASR to evaluate whether functional reactivity to sound was altered in noise-exposed mice. ASR was performed in a small sound-attenuating chamber (IAC) lined with Sonex acoustic foam to reduce acoustic reflections. Mice were placed inside a small, custom-made, sound-permeable, half-cylindrical-shaped testing cage. The testing cage was mounted onto a platform containing a piezoelectric sensor that transduced the animal’s movement into voltage signals that were then amplified using a custom-built amplifier.

All ASR test parameters, stimuli, and recordings were controlled with custom Matlab software. Startle stimuli were 20-ms broadband noise bursts of varying stimulus intensity (70, 80, 90, 100, and 105 dB SPL). Stimuli were generated via a processor (RP2.1; TDT), attenuated (PA5; TDT), amplified (D75A; Crown), and delivered through a speaker (Super Tweeter; Radioshack) placed 10 cm from the testing cage. The speaker was calibrated with a sound level meter (SoundTrack LxT; Larson Davis) in the approximate location of the animal’s head before testing.

Animals were acclimated to the testing cage and sound chamber for 5 min before the start of testing. Each session consisted of 10 trials at each intensity (50 total) presented in pseudo-random order. Trials were separated by a randomized intertrial interval ranging from 5 to 15 s. The animal’s ambient movement was automatically monitored by the testing program at the start of each trial. When the animal was still for a period of 5 s, the startle stimulus was presented. Measurements during the 5-s “quiet movement” period served as a baseline for the animal. For each trial, the animal’s startle response output signals were amplified, digitized, and recorded (RP 2.1; TDT) for 100 ms following the startle stimulus onset. Sessions lasted for 20–25 min. At the end of testing, animals were returned to their home cage. All testing was conducted between the hours of 10:00 am and 4:00 pm by the same experimenter, an adult female.

### Tone detection

We trained mice using operant conditioning to assess how well noise-exposed mice were able to consciously perceive sounds. Mice were trained to detect tones in a quiet background using a conditioned lick suppression (CLS) paradigm (Heffner et al., 2006; Lauer et al., 2011). Because it takes mice several weeks to become proficient at CLS, we began testing within a week after sham or noise exposure. Once mice performed well in the basic task (usually after 3–4 weeks), we began measuring thresholds. During testing, mice were water restricted, but allowed unlimited access to food. On any days they were not tested, we provided mice with supplemental hydrating gel.

Testing took place in a sound-attenuating chamber (IAC) lined in Sonex foam. A mouse was placed in a small wire mesh cage containing a lick spout fed via a syringe pump delivery system. Licking was detected through voltage changes induced when the mouse made contact with the spout. A moist sponge was placed against the bottom of the cage to assist with charge transfer. All testing was controlled by a custom program in Matlab operating a multichannel processor (RX8; TDT). Sounds were produced at a sampling rate of 100 kHz (RX8; TDT) and delivered through an amplifier (SA8; TDT) driving a loudspeaker (Vifa) placed 1 meter from the spout. Before testing, we calibrated tone stimuli using a sound level meter with the tip of the microphone placed at the approximate position of the mouse’s head (Z-weighting; SoundTrack LxT; Larson Davis).

Mice initiated trials by licking the spout. On warning trials, after a random waiting period of between 2 and 5 s, two 240-ms tones separated by a 240-ms silent interval were played from the speaker. A mild shock was presented through the spout 40 ms after sound offset, and mice learned to withdraw from the spout to avoid the shock. Warning trials were presented with a ∼25% probability. During safe trials, no tones were presented, and the animal continued licking to receive juice. Licking was monitored for 720 ms before and after the acoustic stimulus in warning trials and during the equivalent time period for safe trials. For analysis, the relevant time period was split into 35 20-ms “pre” bins and 35 20-ms “post” bins, and we counted the number of bins containing licks. All mice exhibited consistent licking behavior, with licks in at least 20 pre-stimulus bins and no evidence of suppression on safe trials (less than a 10% difference between number of pre- and post-stimulus bins). We considered the mouse to have responded (i.e., suppressed licking) whenever the post-stimulus bin count was less than or equal to the determined criterion. A response on a safe trial was counted as a false alarm, while a response on a warning trial was counted as a hit. The subject’s criterion was calculated such that the false alarm rate was near 16%. Hits and false alarm rates were used to calculate a d′ for each trial type.

In each testing session, we tested mice with tones of one frequency, at levels in 10-dB intervals using the method of constant stimuli and presented in a random order. The range of levels was adjusted to include one level below threshold. Threshold was defined as the level yielding a d′ of 1.0. Given the enforced false alarm rate of 16%, this d′ reflects a hit rate of ∼50%. Mice were allowed to perform trials until they reached satiety, usually 45–60 min, then were returned to their home cages. We measured thresholds for tones of 8, 12, and 16 kHz. Mice were tested with each frequency until the calculated threshold differed by <5 dB for at least three sessions. Final thresholds are reported as the average of the last 3–4 sessions and include at least 20 warning trials at each sound level.

### Spiral ganglion counts

We counted spiral ganglion neurons in the cochleae of our mice to assess the amount of peripheral damage induced by the noise exposure. Cochleae were embedded in Araldite, following methods similar to Hequembourg and Liberman (2001). Mice were deeply anesthetized with a 0.3–0.5 mg/g dose of sodium pentobarbital (i.p.), transcardially perfused with 60 ml of a 4% paraformaldehyde fixative solution, and decapitated. Following transcardial perfusion, we reperfused the cochleae with 4% paraformaldehyde through the round and oval windows and postfixed overnight. Cochleae were then removed from the skull and decalcified in 1% EDTA in phosphate buffer for several days. After a series of progressive dehydrations, cochleae were infiltrated with a solution of Araldite 502 (Electron Microscopy Services) and cured in an oven at 60°C. Cochleae were sectioned parallel to the modiolus at 30 µm and stained with 1% toluidine blue (Fisher Scientific).

We estimated the number of spiral ganglion neurons following stereology procedures described by Schettino and Lauer (2013). We used the Optical Fractionator Probe in Stereo Investigator (SI) software (MBF Bioscience). The region of interest, Rosenthal’s canal, was visualized at 40× magnification and traced. The SI software placed a sampling grid (40 × 40 µm) at random locations over the region of interest. At each counting location, an observer who was blind to the hearing status of the subject counted the number of spiral ganglion neurons within the grid, refocusing as needed to visualize all neurons and measure the total depth of the tissue. The observer continued through all sections that contained Rosenthal’s canal, counting neurons in every third section. When counting was complete, the software estimated the total number of neurons.

### Inner ear immunohistochemistry

To further evaluate the status of the cochlea, we quantified ribbon synapses on inner hair cells and number of inner and outer hair cells in cochlear whole-mount preparations. Cochleae were extracted after transcardial perfusion and fixed for an additional hour, after which they were decalcified in 1% EDTA. We then dissected the organ of Corti into 5–6 flat turns following methods described by Eaton Peabody Laboratories (https://www.masseyeandear.org/research/otolaryngology/investigators/laboratories/eaton-peabody-laboratories/epl-histology-resources/cochlear-dissection-summary). Cochlear pieces were placed in a blocking buffer of 5% normal goat serum, 10% bovine serum albumin, and 0.5% Triton X-100 (Electron Microscopy Services) for 1 h. They were then incubated overnight at 4°C in mouse monoclonal anti-CTBP2 (1:200, BD Biosciences, cat# 612044, RRID:AB_399431), rabbit polyclonal anti-myosin 6 (1:500, Sigma-Aldrich, cat# M5187, RRID:AB_260563), and chicken polyclonal anti-neurofilament (1:1000, Millipore, cat# AB5539, RRID:AB_11212161) in half concentration blocking buffer. The next day, cochlear pieces were rinsed, incubated in secondary antibodies in half concentration blocking buffer for 2 hours at room temperature, rinsed again, mounted in Fluoromount-G (Southern Biotech) on subbed slides, and coverslipped. Secondary antibodies were goat anti-mouse AF488 (1:1000, Thermo Fisher Scientific cat# A-10667, RRID:AB_2534057), goat anti-rabbit AF568 (1:1000, Thermo Fisher Scientific, cat# A-11036, RRID:AB_10563566), and goat anti-chicken AF647 (1:1000, Invitrogen, cat# A21449, RRID:AB_1500594).

We quantified synaptic ribbons and hair cells at 9 frequency locations along the cochlea, falling at half-octave intervals between 4 and 64 kHz. Locations were identified using low-magnification ∼5× photographs and the ImageJ plugin Measure_line, also developed by the Eaton Peabody Laboratories. At each frequency location, we used a confocal microscope (LSM 700 Axio Imager 2; Carl Zeiss) to collect z-stacks at 63× magnification with a z-step size of 0.25 µm. Care was taken to ensure the z-dimension included all synaptic ribbons. Cochlear image processing was done in ImageJ. Synaptic ribbons were calculated as the total number of CTBP2-labeled puncta throughout the image stack divided by the number of inner hair cells in the image. Cells and their ribbons were included only if the entire cell was visible in the image frame. We also counted the total number of inner and outer hair cells that fit within a 100-µm-long window. The number of hair cells in each noise-exposed cochlea was compared to the average number of cells in sham-exposed cochleae to calculate cell survival.

### Ventral cochlear nucleus immunohistochemistry

We evaluated labeling of vesicular glutamate transporter 1 (VGLUT1) and glutamic acid decarboxylase 65 (GAD65) in the VCN to identify effects of noise exposure on excitatory and inhibitory synapses, respectively. VGLUT1 is present in auditory nerve terminals in the cochlear nucleus (CN; Gómez-Nieto and Rubio, 2009; Zeng et al., 2009; Lauer et al., 2013). GAD65 is primarily localized to presynaptic GABAergic terminals (Feldblum et al., 1993; Soghomonian and Martin, 1998); most terminals in the VCN that release GABA also release glycine (Juiz et al., 1996).

Procedures were similar to those described by Lauer et al. (2013) and McGuire et al. (2015). Mice were deeply anesthetized, transcardially perfused, and decapitated, as described above. The skull bone was partially removed, and the brain was postfixed overnight. The following day, the brain was dissected from the skull, trimmed, and embedded in gel albumin. Serial sections were cut at 50 µm in the coronal plane and collected in 0.12 M Tris-buffered saline (TBS).

We incubated sections in 0.5% Triton X-100 (Electron Microscopy Services) for 10 min at room temperature on a shaker. Sections were then blocked in 1% normal goat serum for 1 h at room temperature, rinsed, and incubated overnight in mouse monoclonal anti-GAD65 (1:1000; Abcam, cat# ab26113, RRID:AB_448989) or rabbit polyclonal anti-VGLUT1 (1:1000; Thermo Fisher Scientific, cat# 48-2400, RRID:AB_2533843) at 4°C. The following day, we rinsed the sections, incubated in biotinylated goat anti-mouse (1:200; Vector Laboratories, cat# BA-9200, RRID:AB_2336171) or goat anti-rabbit (1:200; Vector Laboratories, cat# BA-1000, RRID:AB_2313606) for 1 h, rinsed, and then incubated in avidin-biotin complex (ABC Elite, Vector Labs) for 1 h. We finally stained the sections with a solution of nickel ammonium sulfate and 3-3′-diaminobenzidine (Sigma Chemical). For each brain, we omitted the primary antibody for one section and the secondary antibody in an additional section to serve as negative controls. As a positive control, we noted staining of terminals in the cerebellum as has been previously described (Rico et al., 2002; Obata et al., 2008).

### Ventral cochlear nucleus immunohistochemical analysis

Sections that included VCN were photographed in grayscale at 20× on a microscope (Labophot; Nikon) with mounted CCD camera (Progres; Jenoptik). For sections in which VCN did not fit entirely into the imaging area, we took two or three overlapping photographs and merged them in Photoshop (Adobe Systems) or Fiji (Schindelin et al., 2012) before analysis. All photographs of VCN from both hemispheres of a given mouse were then collected as a single image-stack in FIJI. Using a graphics tablet and stylus (Cintiq 22HD; Wacom), we manually traced a region of interest (ROI) around the VCN in each image following standard criteria (Willard and Ryugo, 1983). Each case exhibited minor variations in overall illumination and/or staining intensity across its set of sections. To correct for this irregularity, a leveling adjustment was applied to each section such that the mean pixel intensity within each VCN ROI was equivalent across all sections from the mouse without causing over- or undersaturation. Only global (section-wide) adjustments of pixel values were permitted; local manipulations were never made.

To identify positive immunolabeling, a histogram of pixel intensity was constructed based on all pixels within the VCN ROIs across all slices. This histogram served as the input for automatic image thresholding. We evaluated the output of all available autothreshold algorithms in Fiji against the results of manual thresholding by a blinded observer on a limited dataset. We determined that of the various available algorithms, *triangle* and *RenyiEntropy* most successfully identified stained particles for VGLUT1 and GAD65 labeling, respectively. Furthermore, the algorithms ignored diffuse background labeling such as that noted in negative control sections. Accordingly, these algorithms were applied to our entire dataset. Label/optical density was quantified as the total number of thresholded pixels in a given case divided by the total number of pixels within the entire VCN ROI. With the exception of manual segmentation of VCN borders, all steps were fully automated, thus minimizing the possibility of experimenter bias.

### Cochlear nucleus reconstruction

Each CN was reconstructed and manipulated in 3D following previously described methods (Muniak et al., 2013b; Connelly et al., 2017). Briefly, serial-section bright-field photomicrographs through the entire CN were collected with a 2× objective and semi-automatically montaged and aligned in TrakEM2 software (Cardona et al., 2012) with the assistance of custom Python scripts. Within TrakEM2, the boundary of the CN was segmented using a graphics tablet (Cintiq 22HD; Wacom) following standard criteria (Willard and Ryugo, 1983). We then exported these outlines to Amira software (Thermo Fisher) and used them to construct 3D surfaces of each CN. In instances where the entirety of the CN was not included (e.g., exclusion of posterior-most sections of the DCN), the 3D surface reconstruction was left unbounded (i.e., not “closed”) at the anterior and/or posterior end; doing so ensured that subsequent alignments in Amira were not influenced by artificial boundaries. Each experimental 3D CN surface was iteratively aligned, using affine transformations in Amira, to a published 3D template of the mouse CN (Muniak et al., 2013b) until no further improvements to the quality of the fit could be achieved. We quantified the quality of each fit using the root mean square (RMS) distance of vertices of the experimental surface to the template surface. A 3D transformation matrix was subsequently obtained that provides a linear mapping between an individually reconstructed CN (within the coordinate-frame of TrakEM2) and the CN template. Each hemisphere of a mouse was reconstructed individually, and transformations of the left hemisphere included a reflection over the midline to match the orientation of the CN model.

### Frequency mapping of immunolabeling

Each image section from the 20× image-stack used for immunolabeling analysis was imported into TrakEM2 and aligned to its corresponding 2× counterpart using scale-invariant feature transforms (Saalfeld et al., 2010). The frequency model of the mouse CN (Muniak et al., 2013b) uses the same virtual coordinate system as the aforementioned 3D CN template. Thus, by concatenating the resulting transform of each high-magnification image with the previously computed 3D affine transformation, we were able to map every pixel of the image-stack to the CN frequency model. Accordingly, we could assign frequency values to all pixels within each VCN ROI of a given subject and analyze the results in Matlab. We examined frequency-specific labeling in 1/4-octave bins spanning the frequency range used for audiometric testing (6–32 kHz), with bin-centers of 5.8, 6.9, 8.2, 9.8, 11.7, 13.9, 16.5, 19.6, 23.3, 27.7, and 33.0 kHz. Label density within each bin was evaluated independently: we calculated the total number of immunopositive (i.e., thresholded) pixels—derived from the prior auto-thresholding analysis—within a particular 1/4-octave range and divided this by the total number of VCN pixels allocated to the same frequency interval.

### Experimental design and statistical analyses

We exposed 6-week-old CBA/CaJ mice to broadband noise or a sham exposure. After noise or sham-exposure, mice were used in a variety of procedures; the numbers of mice and their sex and age at the time of procedure are listed in Table 1. We trained some mice in CLS procedures. We do not include ABR data or histology from these animals, because, due to the extensive time spent training and testing them, these other procedures are performed at much later time points. In the rest of the animals, we recorded ABRs 1 month post-exposure. Some of these animals were later tested in ASR, while we collected and processed brains and/or cochleae from others. We did not harvest any tissue from mice tested in ASR due to concern that additional exposure to loud sounds during the ASR testing might have uncontrolled effects on anatomy. Half of the brains collected were labeled for VGLUT1 and half for GAD65; both hemispheres were analyzed in all animals. For most animals, we counted SGNs in both cochleae; however, we did not analyze any cochleae that were damaged during dissection or processing. When two cochleae were available for a given subject, the counts for the two cochleae were averaged to obtain a single count per subject. We dissected one cochlea each from an additional subset of animals and counted ribbon synapses and hair cells.

We fitted linear mixed models (LMM) to all ABR data, as well as ASR data, CLS data, and frequency-specific immunohistochemistry data using the *nlme* package in R (R Development Core Team, 2017) . Mixed models were used because they can account for repeated measures as well as missing data points. Exposure condition and subject sex were included in all models. Additional variables typically included were frequency and stimulus level, but these variables were excluded when not applicable. In general, model fits were quite good, with an average Nagelkerke pseudo-*R*^2^ of 0.75 (Lefcheck, 2016; Nakagawa et al., 2017). Pseudo-*R*^2^ values for the models of ABR thresholds, amplitudes, and latencies were all at least 0.72, except p2 amplitudes in response to tones, which had a value of 0.43. ABR peak ratios of p2:p1 and p4:p1 had models with pseudo-*R*^2^s of 0.50, while that of peak ratio p3:p1 was 0.38. The models for ASR and CLS had pseudo-*R*^2^s of 0.98 and 0.75, respectively, and those for VCN and inner ear immunohistochemistry were >0.82.

We fitted separate models for the ABR thresholds and CLS thresholds, as well as the amplitudes, latencies, and amplitude ratios of each ABR peak. Each model included a weighting term to correct the heteroskedasticity that resulted from differences in variability between the noise-exposed and sham-exposed animals. Amplitude and frequency variables were log-transformed before inclusion in the models to achieve normality, and we also used the *poly* function to include an orthogonal second-order polynomial frequency term to reflect the shape of the audiogram. In the model for ASR, we corrected heteroskedasticity with a weighting term based on stimulus level.

To assess the effect of noise exposure on CLS detection thresholds relative to ABR thresholds, we fit a linear mixed-effects model that included both ABR and CLS thresholds, but also included a “procedure” factor indicating whether a threshold was determined through CLS or ABR. For this analysis, we ignored subject sex due to the smaller number of trained subjects and restricted the data to measurements at frequencies tested in both procedures (i.e., 8, 12, and 16 kHz).

For all mixed-model analyses, the model included all main effects and all possible interaction terms, excluding the highest-order interaction. Statistics reported reflect an ANOVA based on the fixed effects in the model, and we report partial eta squared as an effect size (Cohen, 1973; Richardson, 2011). We performed *post hoc* analyses on the models for thresholds, amplitude ratios, ASR data, and frequency-specific immunohistochemistry data with the package *lsmeans*, using the *mvt* correction (based on a multivariate *t* distribution) for multiple comparisons. We used the *lstrends post hoc* function in the *lsmeans* package to compare the slopes of the level functions for amplitudes and latencies between the noise-exposed and sham-exposed groups, also using the *mvt* correction. For *post hoc* tests, we report Cohen’s *d* as a measure of effect size.

We used two-way ANOVAs to test the effects of noise exposure on spiral ganglion counts and global VCN antibody labeling. ANOVAs were performed using the *ANOVAN* function in Matlab and in all cases included exposure condition and subject sex as factors. We report partial eta squared effect sizes. An alpha level of 0.05 was used for all statistical tests, and residuals from each test followed a normal distribution.

## Results

### Auditory brainstem response

**Figure 1.**
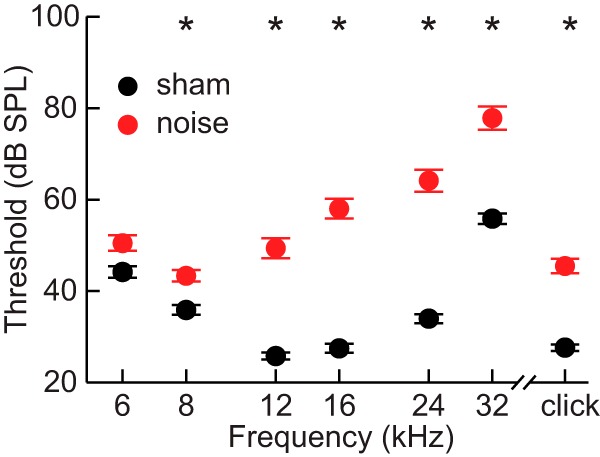
ABR thresholds. Average thresholds for responses to clicks and tones of frequencies ranging from 6 to 32 kHz. Threshold was met when the peak-to-peak amplitude of the response was equal to two standards deviations above the average baseline noise amplitude. Error bars represent SEM. Asterisks indicate significant differences between noise-exposed and sham-exposed subjects.

ABR thresholds were generally higher in noise-exposed mice than in sham-exposed mice (Fig. 1). Thresholds to clicks were significantly higher in noise-exposed animals compared to sham-exposed animals (LMM: *F*_(1,78)_ = 48.8, *p* < 0.001, η_p_^2^ = 0.39), and there was no effect of subject sex. There was a significant effect of the interaction of frequency and exposure condition (LMM: *F*_(2365)_ = 74.0, *p* < 0.001, η_p_^2^ = 0.29) on thresholds in response to tones. Noise-exposed subjects had significantly higher thresholds than sham-exposed subjects in response to all frequencies higher than 6 kHz (all *post hoc*: *t*(78) ≥ 7.2, *p* < 0.001, *d* ≥ 0.80), but the magnitude of the effect was greatest at 16 and 24 kHz. There was also a significant effect of the frequency × sex interaction (LMM: *F*_(2, 365)_ = 5.1, *p* = 0.006, η_p_^2^ = 0.03). There was no difference in thresholds between the sexes at 12 kHz and below, but females tended to have lower thresholds at frequencies above 12 kHz.

**Figure 2.**
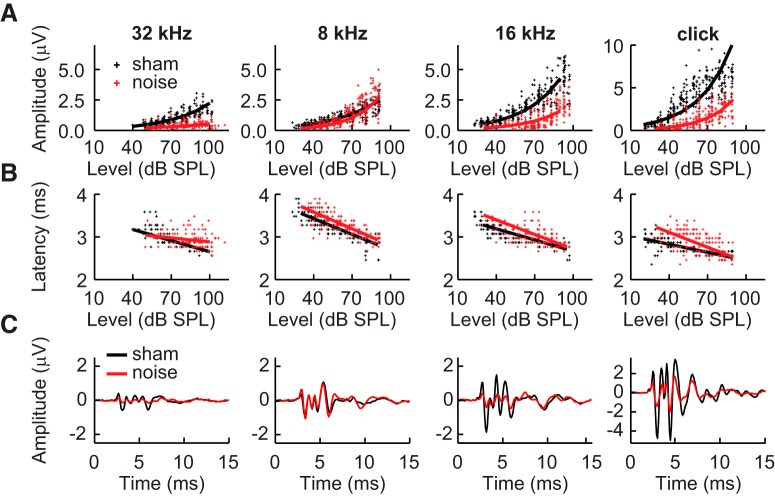
Measurements of ABR peak 1. ***A***, Amplitudes. ***B***, Latencies of peak 1 in response to clicks and tones of 8, 16, and 32 kHz. Legend in ***A*** also applies to ***B***. Axis in ***B*** also applies to ***A***. Solid lines indicate predictions of means made by linear mixed models fit to the data. ***C***, Waveforms averaged across all subjects for each exposure condition. Solid lines represent means, and lighter shading indicates the SEM.

The peak-to-trough amplitudes of ABR wave 1 (referred to hereafter as p1) in response to clicks and tones of 8, 16, and 32 kHz are plotted in Fig. 2*A* (top). Trends for 6, 12, and 24 kHz were similar to those evident in the responses to 8, 16, and 32 kHz, respectively. Amplitudes of p1 in response to clicks and tones of all frequencies increased as a function of intensity (Fig. 2*A*) for both noise-exposed and sham-exposed mice. However, in response to clicks and tones with frequencies above 8 kHz, the amplitudes of p1 were considerably smaller in noise-exposed mice compared to sham-exposed mice (Fig. 2*A*). This point is illustrated by comparing the grand average ABR traces in Fig. 2*C*. The slopes of the level functions were also different. That is, the difference in amplitude between noise-exposed and sham-exposed animals increased as a function of level. This is reflected for the responses to clicks in a significant exposure × level interaction (LLM: *F*_(1390)_ = 6.9, *p* = 0.009, η_p_^2^ = 0.02). For responses to tones, the difference in slopes varied across frequency, as indicated by a significant exposure × frequency × level interaction (LLM: *F*_(2,2368)_ = 44.8, *p* < 0.001, η_p_^2^ = 0.04). The slopes were significantly different between noise-exposed and sham-exposed animals for tones of all frequencies (all *post hoc*: *t*(2368) ≥ 11.8, *p* < 0.001, *d* ≥ 1.3) and clicks (*post hoc*: *t*(390) = 16.0, *p* < 0.001, *d* = 1.77). However, while the slopes were steeper for sham-exposed animals in response to clicks and frequencies from 12 to 32 kHz, they were steeper for noise-exposed animals at 6 and 8 kHz. There was also an effect of sex for some of the level functions for responses to tones, as reflected in a significant sex × frequency × level interaction (LLM: *F*_(2,2368)_ = 6.7, *p* = 0.001, η_p_^2^ = 0.01) and a significant sex × exposure × level interaction (LLM: *F*_(2,2368)_ = 4.6, *p* = 0.033, η_p_^2^ = 0.01). Males had faster slopes than females at 6 and 8 kHz, but females had faster slopes at 12 and 16 kHz; this effect was larger in control mice than exposed mice.

ABR latencies of p1 typically decreased as a function of intensity (Fig. 2*B*), consistent with previous studies in a variety of species (Overbeck and Church, 1992; Zhou et al., 2006; Dehmel et al., 2012a). There was some variation in the slopes of the level functions between noise-exposed and sham-exposed mice and across frequency, reflected in a significant exposure × level interaction for responses to clicks (LMM: *F*_(1390)_ = 38.6, *p <* 0.001, η_p_^2^ = 0.09) and a significant exposure × frequency × level interaction for responses to tones (LMM: *F*_(2,2368)_ = 36.1, *p <* 0.001, η_p_^2^ = 0.03). A *post hoc* test on the slopes indicated differences in noise-exposed and sham-exposed animals for tones at 6, 12, 16, and 32 kHz (all *post hoc*: 4.0 ≤ *t*(2368) ≤ 6.6, *p* <0.001, 0.44 ≤ *d* ≤ 0.73); however, the direction of the differences varied across frequency, and the sizes of the effects were much smaller compared to the effects on amplitudes (compare max latency *d* = 0.73 to min amplitude *d* = 1.30), suggesting that this interaction may not be functionally important. There was also a significant effect of the sex × frequency × level interaction on latencies of responses to tones (LMM: *F*_(2,2368)_ = 10.7, *p <* 0.001, η_p_^2^ = 0.01), but no effects of sex on responses to clicks. The slopes of the level functions were steeper for females than males at 6 and 8 kHz, but did not differ at higher frequencies.

**Figure 3.**
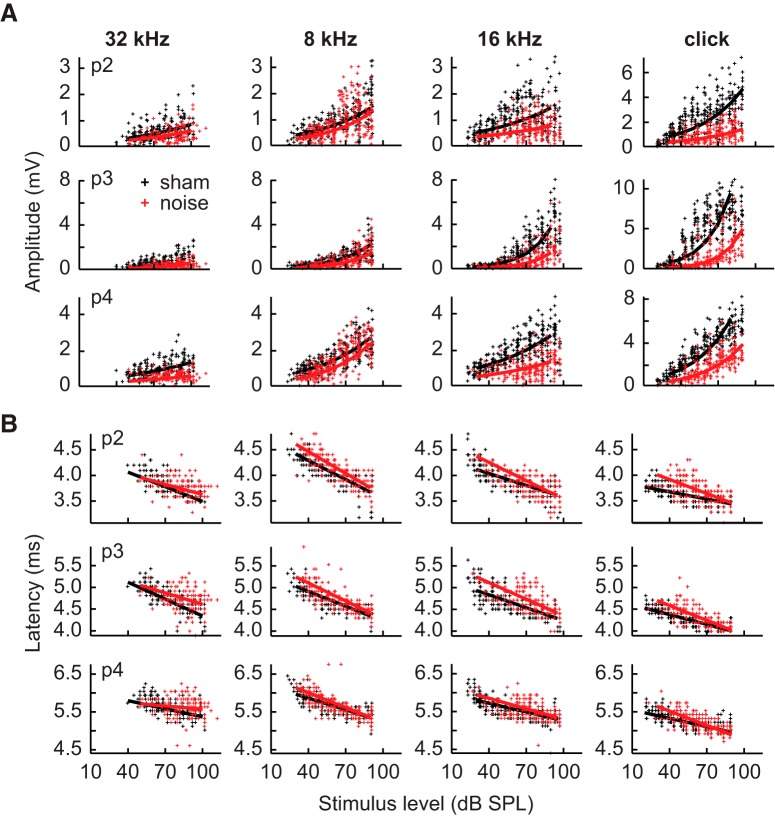
Amplitudes and latencies of ABR peaks 2–4. ***A***, Amplitudes of p2 (top), p3 (middle), and p4 (bottom) in response to clicks and tones of 8, 16, and 32 kHz. ***B***, Latencies for the same corresponding peaks in ***A***. Legend in ***A*** corresponds to all panels. Axis in ***B*** (bottom) corresponds to all panels. Solid lines indicate predictions made by linear mixed models.

Amplitudes of the later ABR peaks (peaks 2–4) increased as a function of increasing level and were generally reduced in noise-exposed animals compared to sham-exposed animals (Fig. 3*A*). There was a significant effect of the exposure × level interaction on amplitudes of responses to clicks for p2 (LMM: *F*_(1390)_ = 3.9, *p* = 0.050, η_p_^2^ = 0.01) and p3 (LMM: *F*_(1390)_ = 6.9, *p* = 0.009, η_p_^2^ = 0.02), but not p4. However, the main effects of exposure (LMM: *F*_(1,78)_ = 29.4, *p* < 0.001, η_p_^2^ = 0.27) and level (LMM: *F*_(1390)_ = 430.0, *p* < 0.001, η_p_^2^ = 0.52) on p4 were both significant. For responses to tones, there were significant effects of the exposure × frequency × level interactions on the amplitudes of p2 (LMM: *F*_(2,2368)_ = 7.4, *p* = 0.001, η_p_^2^ = 0.01) and p4 (LMM: *F*_(2,2309)_ = 19.7, *p* < 0.001, η_p_^2^ = 0.02). None of the three-way interactions had significant effects on p3. However, there were significant effects of the exposure × level (LMM: *F*_(2,2368)_ = 4.7, *p* = 0.031, η_p_^2^ = 0.01), exposure × frequency (LMM: *F*_(2,2368)_ = 5.7, *p* = 0.003, η_p_^2^ = 0.01), and level × frequency (LMM: *F*_(2,2368)_ = 28.711, *p* < 0.001, η_p_^2^ = 0.02) interactions. The slopes of the level functions for p2, p3, and p4 were significantly steeper in sham-exposed animals for clicks and frequencies above 8 kHz (all *post hoc*: *t*(2368) ≥ 4.8, *p* < 0.001, *d* ≥ 0.53) except for p2 at 32 kHz. Slopes for level functions were significantly steeper in noise-exposed animals at 6 kHz for p2 (*post hoc*: *t*(2368) = –5.7, *p* < 0.001, *d* = –0.63) and at 6 and 8 kHz for p4 (*post hoc*: 6 kHz: *t*(2368) = –8.9, *p* < 0.001, *d* = –0.99; 8 kHz: *t*(2368) = –3.5, *p* = 0.002, *d* = –0.39).

Both p2 and p4 of tones were also influenced by a significant effect of the sex × frequency × level interaction (LMM: p2: *F*_(2,2368)_ = 6.1, *p =* 0.002, η_p_^2^ = 0.01; p4: *F*_(2,2368)_ = 3.1, *p =* 0.047, η_p_^2^ = 0.01). There were also significant effects of the sex × frequency × exposure interaction (LMM: *F*_(2,2368)_ = 4.0, *p =* 0.019, η_p_^2^ = 0.01) and the sex × level × exposure interaction (LMM: *F*_(2,2368)_ = 4.2, *p =* 0.042, η_p_^2^ = 0.01) on amplitudes of p2 for responses to tones and of the sex × level interaction (*F*_(1390)_ = 6.1, *p =* 0.014, η_p_^2^ = 0.02) on p4 of responses to clicks. For p2, the slopes for males were not dependent on frequency, but slopes for females decreased as a function of frequency, such that females had steeper slopes than males at low frequencies but shallower slopes at higher frequencies. Slopes for p4 generally decreased in both sexes as a function of frequency, but females had consistently steeper slopes than males across frequency.

Similar trends in latency were observed for the latencies of late peaks as for p1 (c.f. Figs. 2*B* and 3*B*). For all three later peaks of responses to clicks, the interaction between exposure and level was significant (LMM: p2: *F*_(1390)_ = 28.0, *p <* 0.001, η_p_^2^ = 0.07; p3: *F*_(1390)_ = 34.7, *p <* 0.001, η_p_^2^ = 0.08; p4: *F*_(1390)_ = 61.2, *p <* 0.001, η_p_^2^ = 0.14). The interactions between exposure condition, frequency, and level had significant effects on all three of the later peaks of responses to tones (LMM: p2: *F*_(2,2368)_ = 16.8, *p <* 0.001, η_p_^2^ = 0.01; p3: *F*_(2,2368)_ = 13.0, *p <* 0.001, η_p_^2^ = 0.01; p4: *F*_(2,2368)_ = 13.1, *p <* 0.001, η_p_^2^ = 0.01). However, again *post hoc* tests revealed the effects of the exposure × level interactions to be quite small and variable across frequency (all *post hoc*: 2.9 ≤ *t*(2368) ≤ 7.6, *p* ≤ 0.017, 0.44 ≤ *d* ≤ 0.73). The sex × level × frequency interaction was also significant for p3 of responses to tones (LMM: *F*_(2,2368)_ = 5.6, *p* = 0.004, η_p_^2^ = 0.01), reflecting a similar trend as that observed for p1 wherein females had steeper slopes than males only at 6 and 8 kHz.

### Hyperactivity assessed via ABR

**Figure 4.**
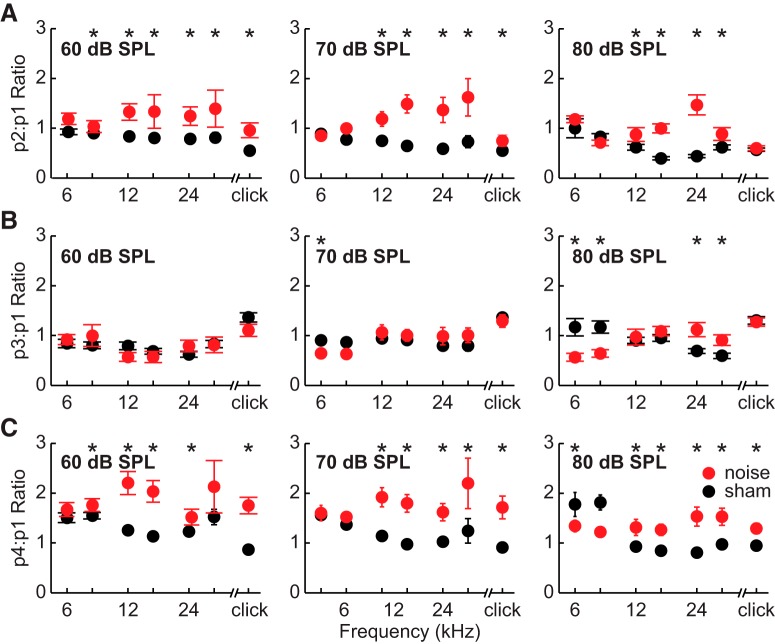
Hyperactivity in bushy cell-driven pathways. Plotted are average ratios of amplitudes of ABR peak 2 relative to peak 1 (***A***), peak 3 relative to peak 1 (***B***), and peak 4 relative to peak 1 (***C***). Ratios are plotted for responses to clicks and tones of frequencies from 6 to 32 kHz at stimulus levels of 60 dB SPL (left), 70 dB SPL (middle), and 80 dB SPL (right). Error bars represent SEM. Legend applies to all panels. Asterisks indicate significant differences between noise-exposed and sham-exposed mice. Ratios larger than those of sham-exposed animals indicate hyperactivity, while smaller ratios indicate hypoactivity.

To evaluate whether the central auditory system exhibited hyperactivity relative to peripheral activity in noise-exposed mice, we calculated the ratio of the amplitudes of peaks 2–4 relative to peak 1 for all responses. The results for stimuli at 60, 70, and 80 dB SPL are plotted in Fig. 4. For responses to clicks, there were significant effects of the exposure × level interaction on p2:p1 (Fig 4*A*; LMM: *F*_(1268)_ = 24.9, *p* < 0.001, η_p_^2^ = 0.09), p3:p1 (Fig 4*B*; LMM: *F*_(1268)_ = 11.4, *p* = 0.001, η_p_^2^ = 0.04), and p4:p1 (Fig 4*C*; LMM: *F*_(1268)_ = 19.5, *p* < 0.001, η_p_^2^ = 0.07) amplitude ratios. No effects that included sex as a variable were significant. We performed *post hoc* contrasts at the stimulus levels 60, 70, and 80 dB SPL. These tests revealed that noise-exposed animals had significantly larger p2:p1 and p4:p1 amplitude ratios than sham-exposed animals, and that the size of the effect was inversely correlated with stimulus level (Table 2).

**Table 2.** *Post hoc* tests comparing click amplitude ratios for noise and sham-exposed mice

Ratio	Level	df	*t*	*p*	*d*
p2:p1	60	178	–5.7	<0.001	–0.63
	70	1.78	–3.1	0.006	–0.34
	80	1.78	–0.1	0.986	–0.02
p3:p1	60	1.78	2.3	0.041	0.25
	70	1.78	1.3	0.269	0.15
	80	1.78	0.3	0.904	0.03
p4:p1	60	1.78	–9.9	<0.001	–1.10
	70	1.78	–7.7	<0.001	–0.85
	80	1.78	–3.5	0.002	–0.39

In responses to tones, there was a general trend for ratios of p2:p1 and p4:p1 to be larger in noise-exposed animals at frequencies of ∼12 kHz and higher, but equal or larger in sham-exposed animals at frequencies below 12 kHz, indicating hyperactivity in the bushy cell–driven pathways in the region of hearing loss. Ratios also differed as a function of level, resulting in significant exposure × frequency × level interactions for each peak ratio (LMM: p2:p1: *F*_(2,1991)_ = 8.2, *p* < 0.001, η_p_^2^ = 0.01; p3:p1: *F*_(2,1991)_ = 19.6, *p* < 0.001, η_p_^2^ = 0.02; p4:p1: *F*_(2,1991)_ = 17.4, *p* < 0.001, η_p_^2^ = 0.01). *Post hoc* tests revealed that noise-exposed animals typically had significantly larger p2:p1 ratios at frequencies of 12 kHz and higher (all *post hoc*: *t*(78) ≥ 2.8, *p* ≤ 0.050, *d* ≥ 0.31). While effect sizes decreased as a function of stimulus level at 12 and 16 kHz (*d* = 0.6–0.39 at 12 kHz and 0.72–0.69 at 16 kHz), they increased as a function of level at 24 and 32 kHz (*d =* 0.60–1.15 at 24 kHz and 0.31–0.98 at 32 kHz). In general, there were no differences between amplitude ratios of noise and sham-exposed mice at 6 or 8 kHz, with the exception of 8 kHz at 60 dB SPL, where noise-exposed mice had larger ratios (*post hoc*: *t*(78) = 3.6, *p* = 0.006, *d* = 0.39).

Most p3:p1 ratios did not differ significantly between noise- and sham-exposed animals. Exceptions occurred almost exclusively at the highest stimulus level examined, 80 dB SPL, and showed frequency dependencies similar to those noted earlier. At 80 dB SPL, noise-exposed mice had larger ratios than sham-exposed mice at 24 kHz (*post hoc*: *t*(78) =3.7, *p* = 0.003, *d* = 0.41) and 32 kHz (*post hoc*: *t*(78) =3.7, *p* = 0.004, *d* = 0.40), but smaller ratios at 6 kHz (*post hoc*: *t*(78) = 5.3, *p* < 0.001, *d* = 0.58) and 8 kHz (*post hoc*: *t*(78) = 3.9, *p* = 0.002, *d* = 0.44). Noise-exposed mice also had smaller ratios at 6 kHz at 70 dB (*post hoc*: *t*(78) = 3.2, *p* = 0.016, *d* = 0.35).

p4:p1 ratios were larger in noise-exposed animals compared to sham-exposed animals at 12 kHz and higher for all levels examined (all *post hoc*: *t*(78) ≥ 3.3, *p* ≤ 0.012, *d* ≥ 0.37), with the exception of 32 kHz at 60 dB SPL where there was no significant difference. Ratios of p4:p1 were also significantly larger in noise-exposed mice than sham-exposed at 8 kHz at 60 dB SPL (*post hoc*: *t*(78) = 4.5, *p* < 0.001, *d* = 0.50), but smaller at 6 kHz at 80 dB SPL (*post hoc*: *t*(78) = 4.7, *p* < 0.001, *d* = 0.51). Similar to p2:p1 ratios, effect sizes of p4:p1 ratios decreased as a function of level at 12 kHz (*d* = 0.77–0.37) and 16 kHz (*d* = 0.83–0.67), but increased as a function of level at 24 kHz (*d* = 0.58–0.95) and 32 kHz (*d* = 0.21–0.73). There were also significant effects of the frequency × level × sex interactions on p2:p1 ratios (LMM: *F*_(2,1991)_ = 17.1, *p* < 0.001, η_p_^2^ = 0.02), p3:p1 ratios (LMM: *F*_(2,1991)_ = 7.2, *p* = 0.001, η_p_^2^ = 0.01), and p4:p1 ratios (LMM: *F*_(2,1991)_ = 15.0, *p* < 0.001, η_p_^2^ = 0.02). For most levels and frequencies, females had larger wave ratios than males, but the differences were small and any observed dependence on level was inconsistent across frequency.

### Acoustic startle reflex

**Figure 5.**
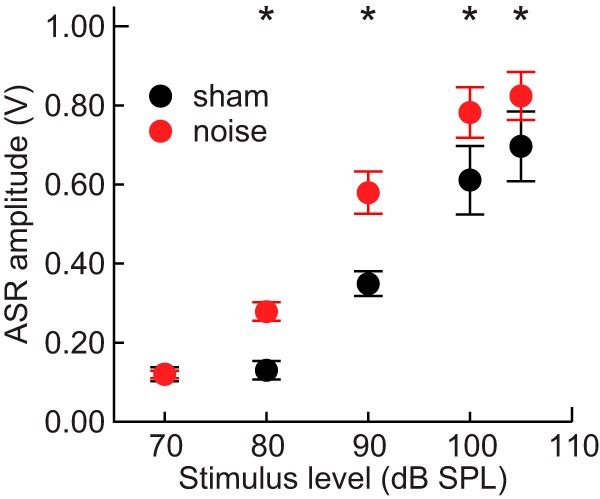
Acoustic startle reflex. Average relative startle responses to broadband noise bursts ranging from 70 to 105 dB SPL. Error bars represent SEM. Asterisks indicate significant differences between groups.

In all animals, a growth in mean ASR amplitude was observed with increasing startle stimulus level (Fig. 5). Mean ASR amplitude was greater for the noise-exposed group compared to the sham-exposed group across all but the lowest stimulus level of 70 dB SPL, which does not typically elicit a startle response. A mixed model revealed a significant effect of the exposure condition × level interaction (LMM: *F*_(1, 81)_ = 14.0, *p* < 0.001, η_p_^2^ = 0.15). Subsequent *post hoc* tests indicated that the effect of noise exposure was significant at all levels except 70 dB SPL (all *post hoc*: *t*(18) ≥ 3.3, *p* ≤ 0.011, *d* ≥ 0.72). Males had larger ASR amplitudes than females at levels above 70 dB SPL, but the size of the effect increased as a function of level, as indicated by a significant sex × level interaction (LMM: *F*_(1,81)_ = 9.6, *p* = 0.003, η_p_^2^ = 0.11).

### Conditioned lick suppression

**Figure 6.**
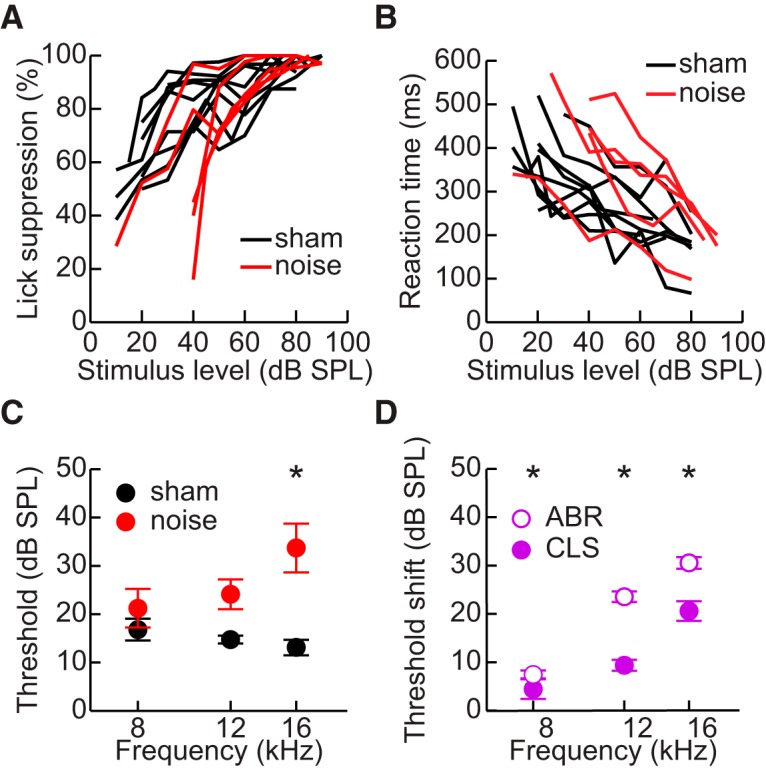
Behavioral detection thresholds. ***A***, Lick suppression rates for noise-exposed and sham-exposed mice for detection of 16 kHz tone. ***B***, Response times for the data in ***A***. ***C***, CLS thresholds for noise-exposed and sham-exposed mice. Error bars indicate SEM. Asterisks indicate significant differences between groups. ***D***, Difference between thresholds of noise-exposed and sham-exposed mice as determined from CLS or ABR. Error bars indicate SEM. Asterisks indicate significant differences between the threshold differences for CLS and ABR.

We measured behavioral detection thresholds to determine if auditory function was maintained in mice that had been noise-exposed. All animals showed lick suppression rates of at least 80% above ∼70 dB (Fig. 6*A*). All animals also showed linear reaction times as a function of stimulus level, with noise-exposed mice typically having longer reaction times (Fig. 6*B*). Noise-exposed animals had higher CLS thresholds than sham-exposed mice, ranging from ∼4.5 dB higher at 8 kHz to ∼20 dB higher at 16 kHz (Fig. 6*C*). There was a significant interaction between frequency and exposure condition (LMM: *F*_(2,32)_ = 6.6, *p* = 0.004, η_p_^2^ = 0.29), with *post hoc* tests revealing a significant difference between noise-exposed and sham-exposed mice only at 16 kHz (*post hoc*: *t*(12) = 2.7, *p* = 0.001, *d* = 1.42).

Importantly, the differences in CLS thresholds were much smaller than the differences in ABR thresholds between noise-exposed and sham-exposed animals, which ranged from ∼8 to ∼30 dB (Fig. 6*D*). A mixed model that included both ABR and CLS thresholds revealed a significant influence of the exposure × procedure interaction (LMM: *F*_(1,92)_ = 8.1, *p* = 0.012, η_p_^2^ = 0.07), indicating that the effect of noise exposure observed through ABRs was significantly different from the effect observed through CLS. *Post hoc* tests revealed that this was true across all three frequencies (all *post hoc*: *t*(92) ≥ 2.8, *p* ≤ 0.006, *d* ≥ 0.29).

### Spiral ganglion neuron counts

Stereological quantification of spiral ganglion neurons indicated that noise-exposed mice experienced a ∼14% loss of SGNs (mean ± SD sham = 7311 ± 595 cells; noise = 6262 ± 717 cells; ANOVA: *F*_(1,15)_ = 10.6, *p* = 0.005, η_p_^2^ = 0.42). There was no significant effect of subject sex. Loss of SGNs would result in a decrease in the input to the central auditory system, consistent with the smaller size observed in ABR peak 1 amplitudes of noise-exposed mice.

### Inner ear immunohistochemistry

**Figure 7.**
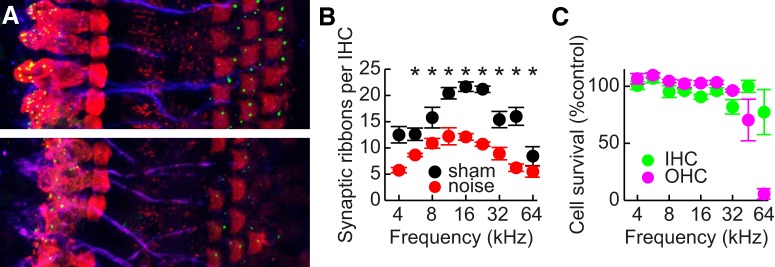
Inner ear immunolabeling. ***A***, Example maximum intensity images of cochleae from a sham-exposed (top) and noise-exposed (bottom) mouse at 32 kHz. Cochleae were immunolabeled for CTBP2 (green), myosin 6 (red), and neurofilament (blue). Scale bar equals 10 µm. ***B***, Average counts of ribbon synapses per inner hair cell as a function of frequency. Error bars indicate SEM. Asterisks indicate significant differences between groups. ***C***, Average percentages of hair cells remaining in noise-exposed cochleae relative to the averages for sham-exposed cochleae. Error bars indicate SEM.

Example images of cochleae labeled for CTBP2 from sham-exposed and noise-exposed mice are shown in Fig. 7*A*. Noise-exposed mice had about half as many ribbon synapses as sham-exposed mice at frequencies between 11 and 45 kHz (Fig. 7*B*). In a linear mixed model, the interaction between frequency and exposure condition was significant (LMM: *F*_(2,56)_ = 3.9, *p* = 0.03, η_p_^2^ = 0.12). The exposure condition had a significant influence on synapse counts at all frequencies except 4 kHz (all *post hoc*: *t*(6) ≥ 3.4, *p* ≤ 0.046, *d* ≥ 1.21). Inner hair cells were well-preserved in noise-exposed mice, with a decrease of ∼20% at 32 and 64 kHz. There was a ∼30% loss of outer hair cells in noise-exposed mice at 45 kHz, with a drastic loss of ∼95% at 64 kHz.

### Ventral cochlear nucleus immunohistochemistry

**Figure 8.**
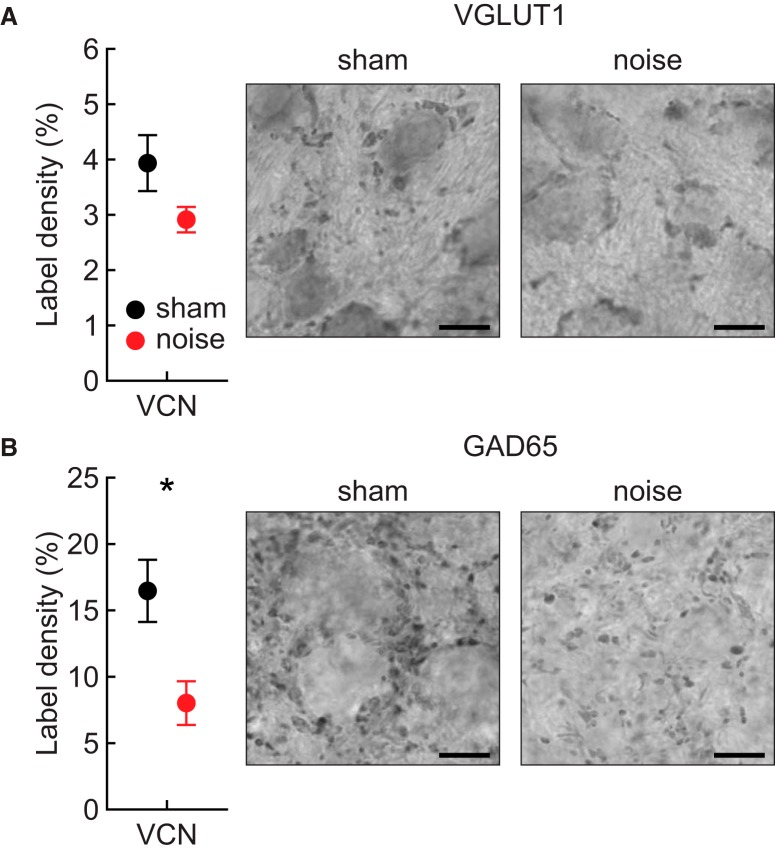
Overall label density in the VCN. ***A***, Quantification of VGLUT1 labeling, calculated as the total number of thresholded pixels divided by the total number of pixels within the entire VCN. Example sections in right panel from a sham-exposed and a noise-exposed individual. Scale bars equal 10 µm. The overall amount of VGLUT1 expression decreased by ∼25% in noise-exposed mice relative to sham-exposed mice, but this reduction was not statistically significant. ***B***, Quantification and examples of GAD65 labeling in sham-exposed and noise-exposed individuals. Noise-exposed animals exhibited a significant reduction of labeling (indicated by asterisk) of ∼50% relative to sham-exposed mice.

**Figure 9.**
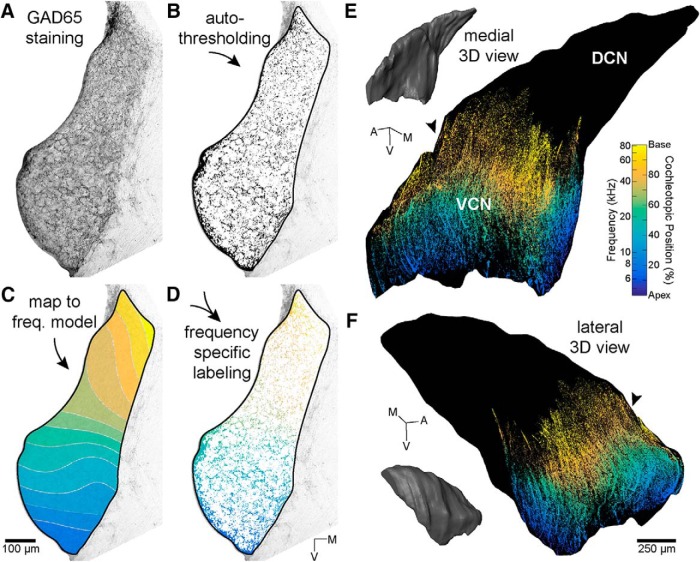
Frequency mapping of immunolabeling. ***A***, An example coronal section of the VCN stained for GAD65. ***B***, The same section in ***A*** following auto-thresholding. Positively-labeled particles are black. The border of the VCN ROI for this section is also shown in black. ***C***, The same section in ***A*** after its corresponding 3D-reconstructed CN was mapped to the frequency model (Muniak et al., 2013b). Colors indicate assigned frequency values. Contour lines indicate edges of 1/4-octave bins used in subsequent analyses. ***D***, Merging of auto-thresholding data (***B***) with frequency mapping (***C***) produces frequency specific GAD65 labeling in the CN. Orientation axes apply to ***A–D***. ***E***, 3D reconstruction of frequency-specific GAD65 labeling for a single case. All coronal slices are shown at their corresponding locations within the reconstructed CN. Black arrowhead indicates position of section shown in panels ***A–D***. The color gradient is a reflection of the tonotopic organization of the VCN. ***F***, Same as in ***E***, but shown from a lateral viewpoint.

Examples of VGLUT1 and GAD65 labeling are shown in Fig. 8. Fig. 9*A*,*B* illustrates an example of the results of autothresholding in a section labeled for GAD65. Across the entire VCN, there was slightly less VGLUT1 labeling in noise-exposed animals compared to sham-exposed animals (Fig. 8*A*). However, this effect was not statistically significant. Noise-exposed mice did have significantly less GAD65 labeling than sham-exposed mice (Fig. 8*B*; ANOVA: *F*_(1,9)_ = 7.4, *p* = 0.024, η_p_^2^ = 0.45). Subject sex had no effect on either type of labeling.

### Frequency distribution of immunolabeling in VCN

To determine if the global changes in VGLUT1 and GAD65 labeling were frequency specific, we reconstructed each nucleus in 3D, fitted the reconstructions to a template of the entire CN (Muniak et al., 2013b), and mapped the frequency information from the template to the reconstruction (Fig. 9*C–F*). The fit quality of the template alignments was similar to that reported previously for mice of the same strain (mean RMS error ± SD 38.6 ± 4.8 μm; Connelly et al., 2017).

**Figure 10.**
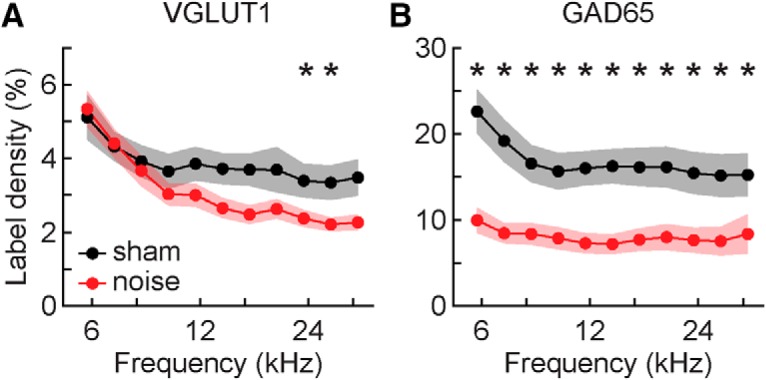
Frequency-specific analysis of immunolabeling. ***A***, Mean density of VGLUT1 expression in the VCN of sham and noise-exposed mice in 1/4-octave bins. ***B***, Mean density of GAD65 expression in the VCN of sham and noise-exposed mice in 1/4-octave bins. Asterisks indicate significant differences following *post hoc* testing.

The difference between VGLUT1 labeling in noise and sham-exposed mice increased as a function of frequency (Fig. 10*A*), reflected in a significant interaction between exposure status and frequency (LMM: *F*_(2114)_ = 28.3, *p* < 0.001, η_p_^2^ = 0.33). *Post hoc* tests revealed significant differences at two of the highest frequencies examined (*post hoc*: 23.3 kHz: *t*(8) = 2.7, *p* = 0.048, *d* = 0.77; 27.7 kHz: *t*(8) = 2.7, *p* = 0.046, *d* = 0.78). While not quite reaching significance after correction for multiple comparisons, differences at the two closest frequencies were of a similar magnitude (*post hoc*: 19.6 kHz: *t*(8) = 2.5, *p* = 0.058, *d* = 0.73; 33 kHz: *t*(8) = 2.6, *p* = 0.054, *d* = 0.75). There was a significant effect of the interaction between sex and frequency (LMM: *F*_(2114)_ = 10.7, *p* < 0.001, η_p_^2^ = 0.16), although the size of this effect was about half that of the exposure status × frequency effect. Females had more labeling at lower frequencies than males, and while labeling decreased in both sexes as a function of frequency, the amount decreased more in females such that they had less labeling than males at the highest frequencies examined. The interaction between exposure status and sex was not significant.

There was no obvious frequency dependence in the differences in GAD65 labeling between noise and sham-exposed mice (Fig. 10*B*). Accordingly, the interaction between exposure status and frequency was not significant, and *post hoc* tests indicated noise-exposed mice had significantly less labeling than sham-exposed mice at all frequencies examined (all: *t*(8) ≥ 3.2, *p* ≤ 0.019, *d* = 0.93). There was also a significant effect of the sex × frequency interaction (LMM: *F*_(2114)_ = 3.6, *p* < 0.030, η_p_^2^ = 0.06). As with VGLUT, GAD65 labeling in both sexes decreased as a function of frequency, but the pattern of the sex difference was reversed. Males had more labeling at the lowest frequencies but less labeling at higher frequencies.

## Discussion

Our experiments demonstrate evidence of hyperactivity in the VCN of mice exposed to damaging noise, which appears to result from a widespread loss of inhibition. While noise-exposed mice had damaged cochleae and elevated ABR thresholds, they showed enhanced reactivity to loud sounds and only slightly elevated behavioral tone detection thresholds. The discrepancy between behavioral and physiologic measures and the imbalance in immunolabeling of excitatory and inhibitory synaptic proteins in the VCN indicate that increased gain in the auditory brainstem can compensate for decreased afferent input to restore or maintain some auditory function.

### Neural substrates of hyperactivity

Noise-exposed mice had elevated ABR wave amplitude ratios compared to sham-exposed mice. Since the late peaks of the ABR are generated by bushy cell–driven pathways (Melcher and Kiang, 1996), elevated wave ratios indicate that these pathways are more responsive than would be predicted given the decreased peripheral input. We observed hyperactivity mostly at frequencies >8 kHz, corresponding to the frequencies where ABR threshold shifts were largest. While this finding is consistent with the general notion that hyperactivity occurs in the central auditory system after sound exposure, most previous studies have limited their reports to later stages of the ascending auditory pathway and the DCN. However, the VCN, where bushy cells are located, is part of several parallel auditory pathways and is involved in basic sound detection and responsivity (Lauer et al., 2017a), so it is perhaps not surprising that hyperactivity is present already in these pathways. Our finding is consistent with two studies demonstrating enhanced spontaneous and driven activity in VCN following noise exposure (Boettcher and Salvi, 1993; Vogler et al., 2011).

Our anatomic results suggest that hyperactivity in VCN results from an overall loss of inhibition, rather than from large changes in excitation. Noise-exposed mice had significantly less GAD65 labeling than sham-exposed mice across all frequencies, but less VGLUT1 labeling only between ∼20 and 30 kHz. The magnitude of the GAD65 decrease coupled with the relative stability of VGLUT1 labeling represents an imbalance between inhibition and excitation in the brainstem that likely accounts for the observed hyperactivity. There was not a strong effect of frequency on any of our results, which is likely due to the broadband character of the exposure. Other authors exposing with different types of noise have found different patterns of hearing loss (Chen et al., 2016).

Our VGLUT1 labeling is consistent with that of Kurioka et al. (2016), who showed that VGLUT1 puncta density decreased in the VCN of noise-exposed mice. Furthermore, studies in a variety of animal models have shown a loss of VGLUT1 in VCN after treatment with ouabain or kanamycin, conductive hearing loss, and mechanical hair cell ablation (Zeng et al., 2009; Fyk-Kolodziej et al., 2011; Yuan et al., 2014; Clarkson et al., 2016). The results of our GAD65 labeling contrasts with that of Zheng et al. (2015), who found no difference in GAD labeling in the VCN of noise-exposed and control rats. However, the rats in the Zheng et al. study did not develop permanent ABR threshold shifts, probably because of differences in exposure protocol and species-specific susceptibility to noise exposure. Furthermore, Zheng et al. (2015) evaluated GAD expression with an antibody against both GAD65 and GAD67, while the current study targeted only GAD65. The two isoforms tend to localize differently in cells (Soghomonian and Martin, 1998), so it is not surprising that they might be differentially reorganized following sound exposure. It is worth noting that GAD65 in the VCN is likely localized mostly to terminals that co-release glycine and GABA. While glycine is typically considered the primary inhibitory neurotransmitter in the VCN (Wu and Oertel, 1986), GABA predominantly acts to modulate timing (Palombi and Caspary, 1992; Gai and Carney, 2008; Chanda and Xu-Friedman, 2010) and primary inhibition (Lim et al., 2004; Lu et al., 2008; Nerlich et al., 2014). Other forms of peripheral damage are also associated with decreases in inhibitory protein immunolabeling and concentrations of inhibitory amino acid in the VCN (Syka, 2002; Asako et al., 2005; Godfrey et al., 2005, 2008; Lu et al., 2008; Gold and Bajo, 2014).

### Central hyperactivity as compensation

Despite large increases in ABR thresholds, noise-exposed mice did not suffer large behavioral deficits. In fact, noise-exposed mice showed increased reactivity to acoustic startle-inducing stimuli, similar to previous reports (Chen et al., 2013; Salloum et al., 2014). Salloum et al. (2014) showed that increased in ASR reactivity was dependent on a moderate elevation in ABR thresholds, which explains why some other experiments did not identify large differences in the ASR after sound exposure (e.g. Sun et al., 2012; Hickox and Liberman, 2014).

The elevation in ASR amplitudes suggests that sound-exposed subjects are sensitized to loud sounds. Sound-exposed rats show evidence of increased stress or anxiety (Pace and Zhang, 2013; Lauer et al., 2017b), and emotional states can potentiate the ASR (Davis et al., 1993; Lauer et al., 2017a). Although we did not test for stress, it is possible that the noise-exposed mice in our study experienced negative emotional states a result of the exposure. The exaggerated ASR could additionally be related to the emergence of tinnitus or hyperacusis in our noise-exposed subjects (see Impairments related to central hyperactivity, below). However, we did not try to determine if any of our animals had either tinnitus or hyperacusis. Regardless of additional factors, the startle-eliciting stimulus was sufficient to elicit robust ASRs in noise-exposed mice, providing further support that central gain was able to compensate for reduced peripheral input.

Though ASR measures are informative about reactivity to sounds, they do not necessarily reflect conscious perception, since the animal is not required to attend to the stimuli (Lauer et al., 2017a). In a trained behavioral task, our noise-exposed mice had nearly normal detection thresholds, indicating that perception was not as severely impaired as the ABR thresholds and SGN loss might suggest. These results are consistent with data from aging gerbils, noise-exposed rats, noise-exposed chinchillas, and ouabain-treated mice, in which electrophysiological measures of threshold did not reliably estimate behavioral thresholds after acquired hearing loss (Salvi et al., 1979; Hamann et al., 2002; Heffner et al., 2008; Chambers et al., 2016).

One could argue that ABR thresholds are higher than behavioral thresholds in mice with peripheral trauma because the ABR is a measure of synchronous neural activity and may require coordination of larger numbers of neurons than perception does. However, Ngan and May (2001) showed that ABR thresholds correlate with the thresholds of the single most sensitive auditory nerve fibers in subjects with noise-induced hearing loss, suggesting that the ABR thresholds are not artificially inflated but instead accurately reflect the physiologic status of the auditory nerve after hearing loss.

We suggest that noise-exposed mice preserve or regain some auditory function through compensation in the central auditory system. Several studies have demonstrated increased central gain in response to peripheral damage (Salvi et al., 2003; Gold and Bajo, 2014; Chambers et al., 2016; Clarkson et al., 2016; Möhrle et al., 2016; Zhao et al., 2016), although the underlying mechanisms remain uncertain (Auerbach et al., 2014). This increase in gain is thought to partially compensate for the reduced sensory input. Typically, the compensation is thought to begin in the DCN, inferior colliculus, or auditory cortex. In the present study, we observed hyperactivity in bushy cell-driven pathways, suggesting that compensation is already present at the level of the brainstem, at least under the conditions tested here. Mice unilaterally treated with ouabain (Chambers et al., 2016) provide an interesting counterpoint, because when tested only with the treated ear, they show normal behavioral detection thresholds, virtually no ASR, and very little ABR. The mice exhibit hyperactivity at the level of the auditory cortex, with some elevation in the inferior colliculus. Ouabain eliminates nearly all type I SGNs and their central processes (Lang et al., 2005), but other forms of peripheral damage usually spare most SGNs and their central processes (Zilberstein et al., 2012; McGuire et al., 2015). This difference in SGN survival likely accounts for the lack of startle response in the ouabain-treated animals and may explain why compensation is strongest at the level of the cortex. Nevertheless, our experiments demonstrate that gain compensation is possible at the first level of the central auditory pathway.

### Impairments related to central hyperactivity

Central hyperactivity may be able to compensate for the loss of afferent input from the ear, and thus may represent a potential target for novel treatments. However, it has also been associated with various forms of hearing dysfunction. Increased central ABR wave amplitudes relative to wave 1 have been implicated in tinnitus and reduced sound tolerance in some human studies (Attias et al., 2005; Gu et al., 2010; Schaette and McAlpine, 2011), although not in others (Møller et al., 1992; Gilles et al., 2016). In animal studies, central hyperactivity has been associated with tinnitus, reduced sound tolerance, and loudness recruitment (Attias et al., 2005; Cai et al., 2009; Middleton et al., 2011; Heeringa and van Dijk, 2014; Kalappa et al., 2014). Tinnitus and reduced sound tolerance are associated with impaired speech comprehension in noise (Vielsmeier et al., 2016) and can cause difficulty using amplification devices (Lindley et al., 2001; Formby et al., 2008). Hyperactivity in central auditory pathways could affect other aspects of perception such as spectral contrast perception and temporal processing (Møller, 1972; Blackburn and Sachs, 1990; Young et al., 1992; Doucet et al., 1999; McGinley and Oertel, 2006; Chen et al., 2016). Central hyperactivity may also result in abnormal activation of the olivocochlear pathways (Knudson et al., 2014; Dragicevic et al., 2015), which could further reduce the afferent input to the brain and affect hearing in noise and selective attention (Delano et al., 2007; Sturm and Weisz, 2015). Thus, while detection of sounds may be restored by central hyperactivity, more complex aspects of hearing may be adversely affected. While we did not test our subjects for tinnitus or sound tolerance, some subset of our subjects may have experienced one or more of these additional pathologies. Identification of these subgroups in future studies (following, e.g., Gu et al., 2010; Hickox and Liberman, 2014) may reduce variability in results and inform our understanding of these pathologies. When developing therapies and optimizing prosthetic devices, it is important to consider how the central auditory system may compensate for peripheral changes.

## Synthesis

Reviewing Editor: Christophe Bernard, INSERM & Institut de Neurosciences des Systèmes Decisions are customarily a result of the Reviewing Editor and the peer reviewers coming together and discussing their recommendations until a consensus is reached. When revisions are invited, a fact-based synthesis statement explaining their decision and outlining what is needed to prepare a revision will be listed below. Note: If this manuscript was transferred from JNeurosci and a decision was made to accept the manuscript without peer review, a brief statement to this effect will instead be what is listed below.

The authors did address many of the technical criticisms raised by the Journal of Neuroscience reviews.

However, as noted by one very expert reviewer:

“Methods are excellent, the results are convincing but for the most part, the results are predictable and similar to material already in the literature.”

I carefully read the revised Ms and must agree. There is not sufficient new insights in this manuscript to warrant publication in a general neuroscience journal like eNeuro. This would find a more suitable home in a journal specialized for audtory system research.
